# Insects as an Alternative Protein Source: A Sustainable Approach to Future Food Security

**DOI:** 10.3390/insects17060655

**Published:** 2026-06-22

**Authors:** Mohd Suhail Banday, Ambashree Dubey, Neha Thakur, Saima Banday, Jyoti Jawla, Jameel Ahmad, Esteban Pérez-García, Ariana Saraiva, Hmidan A. Alturki, António Raposo

**Affiliations:** 1 Division of Livestock Products Technology, ICAR–Indian Veterinary Research Institute, Bareilly 243122, India; ranudubey443@gmail.com (A.D.); jawlajyoti@gmail.com (J.J.); nig.jam786@gmail.com (J.A.); 2 Department of Livestock Products Technology, Lala Lajpat Rai University of Veterinary and Animal Sciences, Hisar 125004, India; nehathakur@luvas.edu.in; 3 Department of Veterinary Medicine, Sher-e-Kashmir University of Agricultural Sciences & Technology of Jammu, Jammu 181102, India; saimabanday22@gmail.com; 4 Department of Animal Pathology and Production, Bromatology and Food Technology, Faculty of Veterinary, Universidad de Las Palmas de Gran Canaria, Trasmontaña s/n, 35413 Arucas, Spain; esteban.perezgarcia@ulpgc.es; 5 Research in Veterinary Medicine (I-MVET), Faculty of Veterinary Medicine, Lisbon University Centre, Lusófona University, Campo Grande 376, 1749-024 Lisboa, Portugal; ariana.saraiva@ulusofona.pt; 6 Veterinary and Animal Research Centre (CECAV), Faculty of Veterinary Medicine, Lisbon University Centre, Lusófona University, Campo Grande 376, 1749-024 Lisboa, Portugal; 7 King Abdulaziz City for Science & Technology, Wellness and Preventive Medicine Institute—Health Sector, Riyadh 11442, Saudi Arabia; halturki@kacst.edu.sa; 8 CBIOS (Research Center for Biosciences and Health Technologies), ECTS (School of Health Sciences and Technologies), Lusófona University, Campo Grande 376, 1749-024 Lisboa, Portugal

**Keywords:** alternative protein, consumer acceptance, edible insects, environmental impact, entomophagy, food security, nutrition, sustainability

## Abstract

Edible insects are increasingly recognized as a viable alternative protein source, offering a potentially sustainable approach to addressing global food security challenges. This narrative review critically examines the nutritional composition, environmental advantages, techno-functional attributes, and potential applications of insect-based proteins within human food systems. Edible insects are characterized by high protein content, favourable essential amino acid profiles, and appreciable levels of key micronutrients, rendering them nutritionally comparable to conventional livestock-derived proteins. Moreover, insect production systems generally require substantially lower inputs of land, water, and feed, resulting in comparatively lower greenhouse gas emissions and reduced overall environmental burden. Despite these advantages, broader adoption remains constrained by challenges related to regulatory heterogeneity, food safety concerns, and limited consumer acceptance. Overall, the available evidence suggests that edible insects can function as a nutritionally adequate and environmentally sustainable complementary protein source; however, significant variability in nutrient composition, limitations in standardized safety assessment, and socio-cultural barriers currently restrict their large-scale integration into mainstream food systems. In addition, inconsistencies in analytical methodologies and reliance on in vitro data further complicate cross-study comparisons and translational relevance. Future research should focus on standardization of rearing and processing conditions, harmonization of evaluation frameworks (e.g., protein quality indices), comprehensive safety assessments, and well-designed clinical studies to validate nutritional and functional benefits, alongside the development of effective strategies to improve consumer acceptance and support regulatory alignment across regions.

## Simple Summary

Global demand for sustainable protein sources is increasing as food systems face growing environmental and resource constraints. Edible insects have emerged as promising alternatives because they provide high-quality protein, essential nutrients, and can be produced with lower land, water, and feed requirements than conventional livestock. This review provides an updated and integrated overview of edible insects by examining nutritional quality, protein digestibility, processing technologies, safety considerations, consumer acceptance, regulatory developments, and commercial applications. Recent advances in insect farming, nutrient-enrichment strategies, and insect-derived functional ingredients are also discussed. Although edible insects show considerable potential to support sustainable food and feed systems, challenges related to standardization, allergenicity, regulatory harmonization, and consumer acceptance remain important barriers to wider adoption.

## 1. Introduction

The global food system is under growing pressure to meet rising protein demand while minimizing environmental impacts. According to projections by the United Nations, the global population is anticipated to reach approximately 9.7 billion by 2050, peak at around 10.3 billion in the 2080s, and then slightly decline to about 10.2 billion by 2100 [1]. Meeting the nutritional needs of this growing population will require substantial increases in food production, with some estimates suggesting that output may need to nearly double by mid-century [2], thereby intensifying pressure on already constrained natural resources. This challenge is not only quantitative but also qualitative, because future protein systems must provide adequate nutrition while remaining environmentally and economically sustainable.

Conventional livestock production currently supplies a major proportion of global dietary protein; however, it is associated with considerable environmental burdens, including extensive land and water use, deforestation, high energy demand, and significant greenhouse gas emissions [3]. These constraints highlight the limitations of existing protein production systems and underscore the urgency of identifying sustainable and resource-efficient alternatives. In this context, alternative protein sources are receiving increasing attention as part of broader efforts to develop resilient food systems.

Edible insects have attracted attention as a viable alternative protein source owing to their favourable nutritional profile and efficient production [4]. Insects are valuable sources of high-quality protein, essential amino acids, vitamins, minerals, and other bioactive compounds and have been traditionally consumed in many regions worldwide [5]. Compared with conventional livestock systems, insect farming generally requires fewer natural resources and may generate lower environmental impacts while supporting circular-bioeconomy approaches through the valorization of organic side streams [6]. In addition, insect production systems are scalable and adaptable, making them suitable for both small- and large-scale operations. Despite these advantages, their integration into modern food systems remains limited due to challenges related to standardization, safety, allergenicity, regulatory frameworks, and consumer acceptance. Furthermore, the nutritional composition, protein quality, digestibility, and techno-functional properties of edible insects vary considerably according to species, developmental stage, feed substrate, rearing environment, and processing conditions, which complicates direct comparisons among studies and limits the generalization of findings.

Numerous reviews have examined specific aspects of edible insects, including nutritional composition, protein quality, processing technologies, allergenicity, safety, consumer acceptance, and regulatory considerations [7,8,9]. Collectively, these studies have substantially advanced understanding of insects as alternative protein sources. However, most reviews have addressed these dimensions separately, whereas recent developments in industrial-scale production technologies, protein quality assessment methodologies, allergenicity mitigation strategies, precision insect farming, and commercialization have generated new evidence that has not yet been comprehensively integrated. As a result, there remains a need for an updated and multidisciplinary assessment that connects nutritional quality, processing effects, safety considerations, consumer acceptance, and market feasibility within a single analytical framework.

In addition, limited attention has been given to the combined evaluation of protein quality metrics such as the Protein Digestibility-Corrected Amino Acid Score (PDCAAS) and the Digestible Indispensable Amino Acid Score (DIAAS), processing-induced modifications affecting nutritional functionality, and allergenicity mitigation strategies within a unified framework [8]. Furthermore, inconsistencies in methodological approaches and the continued reliance on in vitro evidence for many proposed health-promoting properties complicate direct comparisons and reduce translational applicability. These limitations make it difficult to identify which insect species, production systems, and processing approaches are most promising for food applications.

Recent advances in controlled-environment insect farming, automation, artificial intelligence, genomic tools, precision feeding, nutrient biofortification, and insect-derived functional ingredients have further expanded the potential applications of insects beyond traditional protein production. At the same time, evolving regulatory frameworks and increasing commercial interest have created new opportunities and challenges that were not comprehensively addressed in many earlier reviews. These developments highlight the need for a critical synthesis of current knowledge that moves beyond descriptive accounts of nutritional composition and sustainability benefits.

Therefore, this review examines the nutritional value, environmental and economic relevance, processing technologies, consumer acceptance, safety considerations, regulatory developments, and future prospects of edible insects as alternative protein sources. Particular attention is given to recent advances in protein quality evaluation, processing-induced modifications, allergenicity mitigation strategies, precision insect farming, and the development of insect-derived bioactive compounds. By integrating these dimensions within a unified framework, the present review aims to provide an updated and application-oriented perspective on edible insects and their potential contribution to sustainable food and feed systems. Ultimately, the review seeks to identify the major scientific, technological, regulatory, and market challenges that must be addressed for edible insects to contribute meaningfully to future food security and sustainable nutrition.

## 2. Methods

This review was conducted using a narrative literature review approach to provide an updated and integrated overview of edible insects as alternative protein sources for food and feed applications.

Relevant literature was identified through searches of major scientific databases, including Scopus, Web of Science, PubMed, ScienceDirect, and Google Scholar. The literature search was conducted between January and March 2025. Search terms included combinations of keywords such as “edible insects”, “insect protein”, “entomophagy”, “alternative protein sources”, “food security”, “sustainability”, “nutritional composition”, “protein quality”, “consumer acceptance”, “food safety”, “processing technologies”, “regulatory frameworks”, and related terms.

Priority was given to peer-reviewed journal articles published in English between 2015 and 2025 to ensure that the review reflects current scientific evidence, technological developments, industrial trends, and regulatory advances. However, seminal earlier publications and authoritative reports from international organizations were also included when considered relevant to provide historical context or foundational knowledge.

Publications were selected based on their relevance to one or more of the following topics: nutritional composition and protein quality, bioavailability, environmental and economic sustainability, insect farming and production systems, processing technologies, food safety and allergenicity, consumer perception and acceptance, regulatory developments, commercialization, and emerging applications of edible insects. Conference abstracts without full-text availability, non-English publications, and sources lacking sufficient scientific or methodological information were generally excluded.

The selected literature was critically examined and organized into thematic sections addressing nutritional value, environmental and economic benefits, edible insect species and consumption patterns, processing technologies, consumer acceptance, safety and regulatory considerations, commercially available products, and future prospects. The aim was not to perform a systematic review or meta-analysis, but rather to provide a comprehensive and multidisciplinary synthesis of the current knowledge and emerging developments regarding the role of edible insects in sustainable food systems.

## 3. Nutritional Value of Edible Insects

Edible insects have received increasing attention as alternative protein sources due to their favourable nutritional profiles, which include high protein content, well-balanced amino acid profiles, beneficial lipid components, and significant levels of essential micronutrients [10]. At the same time, their nutrient profile is far from uniform, and it can change markedly with species, developmental stage, diet and processing conditions. This variability remains one of the main obstacles to standardization and to the wider use of insects in food systems.

### 3.1. Protein Content, Digestibility, and Amino Acid Quality

On average, the protein content of edible insects typically ranges from approximately 35% to 75% on a dry weight basis (or about 10–25% on a fresh weight basis) [11,12]. Many species can match and, in some cases, even exceed, the protein levels of conventional sources such as cereals, legumes, beef and poultry when compared on the same basis [13,14]. However, this broad range reflects not only biological diversity but also differences in analytical methods, making direct comparisons across studies difficult.

In addition to protein quantity, insect-derived proteins are generally regarded as nutritionally valuable, with in vitro protein digestibility values commonly reported in the range of approximately 77% to 90% and in some cases reaching up to around 95% depending on species and processing conditions [15]. Even so, in vitro values should be interpreted carefully, because they do not always reflect how the protein will behave in the human digestive tract. Factors such as chitin content, heat treatment and matrix effects can all influence actual protein utilization.

Furthermore, edible insects provide a balanced amino acid profile rich in essential amino acids, with indispensable amino acids often accounting for more than 40% of the total amino acid content, supporting their potential as a high-quality protein source [16,17,18]. Most edible insect species meet or come close to human amino acid requirements, although some species may show slight limitations in sulphur-containing amino acids, especially methionine and cysteine [19,20]. This suggests that insects can be nutritionally strong ingredients, but they may still benefit from protein complementation or processing adjustments when used in specific food formulations.

Among different taxonomic groups, Orthoptera such as crickets, grasshoppers and locusts often show particularly high protein levels, commonly around 61% to 77% on a dry-matter basis [21]. The variation in protein content across major edible insect groups is summarized in Table 1. Likewise, species such as *Cirina butyrospermi* and *Musca domestica* have been reported to contain crude protein levels of around 60% to 64% of dry matter, which is comparable to values reported for conventional animal protein sources [22]. Still, these should not be treated as fixed species values, because protein content can shift substantially with diet, growth stage and processing method.

Overall, Table 1 demonstrates considerable variation in protein content among edible insect species, with values ranging from approximately 20% to 77% on a dry matter basis. Orthopteran species, including crickets and locusts, generally exhibit higher protein concentrations, whereas termites and some dipteran larvae display greater variability. These differences may be attributed to species-specific biological characteristics, feeding habits and nutrient storage patterns. Moreover, the broad protein ranges reported for individual species suggest that nutritional composition is influenced not only by taxonomy but also by environmental and production-related factors. Therefore, protein values reported for edible insects should be regarded as variable rather than fixed characteristics. This variability highlights the importance of considering both species selection and production conditions when evaluating insects as alternative protein sources.

However, protein content and quality can vary considerably depending on species, developmental stage, diet and processing methods [4]. Variations across life stages in edible insects have been observed, with late instar larvae and nymphs often showing protein levels comparable to or higher than adults; however, diet, rearing conditions and species-specific factors are generally more influential than developmental stage alone [25]. Feed substrate composition is particularly important, as differences in dietary ingredients can significantly affect crude protein content, amino acid composition and protein digestibility [26]. Studies have shown that the use of different feed sources, including agricultural by-products and organic side streams, can result in substantial variation in the nutritional quality of insect biomass. Consequently, comparisons among studies should be made with caution, especially when insects have been reared under different feeding regimes. Sex-related differences are generally less pronounced, although females of some species may contain slightly higher protein levels due to physiological processes associated with reproduction [27]. In contrast, essential amino acid profiles tend to remain relatively stable between sexes, suggesting that overall protein quality is less affected by biological sex than by environmental and nutritional factors [16,20]. Collectively, these findings indicate that rearing and feeding conditions are among the primary determinants of protein variability in edible insects.

An additional challenge when comparing protein values across studies is the lack of methodological standardization. Protein content is commonly estimated using nitrogen-to-protein conversion factors; however, insects contain non-protein nitrogen compounds, particularly those associated with chitin, which may lead to an overestimation of true protein content when conventional conversion factors are used [27]. For this reason, several authors have recommended the use of insect-specific conversion factors to improve the accuracy and comparability of protein measurements. Such methodological differences should be taken into account when interpreting protein data reported in the literature.

Finally, although high digestibility and favourable amino acid profiles are frequently reported, variations associated with processing methods (e.g., drying, roasting and defatting) highlight the need for standardized evaluation approaches. Processing can improve protein digestibility by disrupting structural barriers and enhancing protein accessibility; however, excessive heat treatment may reduce amino acid availability through protein denaturation and Maillard reactions [28,29]. The application of robust protein quality indices, such as PDCAAS and DIAAS, may provide more reliable comparisons across studies and support the accurate assessment of insect-derived proteins for food and feed applications [13,30,31]. Nevertheless, inconsistencies in analytical methodologies, limited in vivo validation and the absence of universally accepted evaluation protocols remain important constraints in current protein quality assessment frameworks.

### 3.2. Lipids, Vitamins, and Minerals

In addition to protein, edible insects contain substantial amounts of lipids, typically contributing approximately 10–50% on a dry matter basis, depending on species, developmental stage, and diet [4,32,33]. These lipids are predominantly composed of unsaturated fatty acids, including considerable proportions of monounsaturated (MUFAs) and polyunsaturated fatty acids (PUFAs), with commonly reported fatty acids such as oleic acid (C18:1), linoleic acid (C18:2n − 6), and, in some species, α-linolenic acid (C18:3n − 3) [4,32]. This lipid composition suggests potential nutritional advantages, particularly in terms of essential fatty acid intake and cardiovascular health relevance.

Certain species, such as *Cirina butyrospermi* and various grasshoppers and caterpillars, have been reported to contain relatively high levels of PUFAs, which may contribute to dietary essential fatty acid intake, particularly in regions where conventional lipid sources are limited [32]. However, the fatty acid profile remains highly dependent on feed substrate and rearing conditions, indicating opportunities for nutritional modulation through controlled production systems. Studies have demonstrated that the fatty acid composition of edible insects can be altered through dietary manipulation, creating opportunities for the enrichment of beneficial fatty acids and the production of insect-derived ingredients with improved nutritional quality.

Despite their nutritional advantages, the relatively high proportion of unsaturated fatty acids may also present technological challenges. Polyunsaturated fatty acids are particularly susceptible to oxidative degradation during processing and storage, which can result in lipid rancidity, off-flavour development and reduced nutritional quality. Consequently, appropriate preservation strategies, including controlled drying conditions, oxygen-limiting packaging systems and refrigerated storage, may be necessary to maintain the quality and shelf-life of insect-based products. These considerations are especially important for products intended for long-term storage or large-scale commercialization.

Edible insects also provide a wide range of essential micronutrients, often at levels comparable to or higher than those reported for conventional animal-derived foods [4,32,33]. These minerals can be broadly categorized into macro-minerals and microminerals based on their physiological requirements. Macro-minerals, including calcium (Ca), phosphorus (P), magnesium (Mg), potassium (K) and sodium (Na), play important roles in skeletal development, electrolyte balance, nerve transmission and energy metabolism. Microminerals or trace elements, including iron (Fe), zinc (Zn), copper (Cu), manganese (Mn) and selenium (Se), are required in smaller quantities but are essential for immune function, antioxidant defence, oxygen transport and numerous enzymatic processes.

Several edible insect species have been reported to contain substantial amounts of iron and zinc, in some cases exceeding concentrations reported for conventional livestock products. For example, mopane worms (*Gonimbrasia belina*) and certain orthopteran species have been identified as particularly rich sources of bioavailable iron and zinc, suggesting potential value in addressing micronutrient deficiencies in vulnerable populations [26,34,35]. Among the most frequently reported vitamins are riboflavin (B_2_), pantothenic acid (B_5_), biotin (B_7_), folate (B_9_) and vitamin B_12_, all of which play important roles in cellular metabolism, nervous system function and haematopoiesis [4,32]. In several species, the concentrations of selected B-complex vitamins have been reported to be comparable to or higher than those found in conventional meat products, highlighting their potential contribution to dietary micronutrient intake. Furthermore, recent studies suggest that vitamin concentrations in edible insects may be influenced by feed composition and rearing conditions, creating opportunities for nutritional enhancement through targeted feeding strategies.

These micronutrient profiles indicate that edible insects may contribute to addressing micronutrient deficiencies in specific populations. Unlike conventional livestock products, where muscle tissue is primarily consumed, edible insects are often consumed whole. Consequently, consumers may benefit from nutrients distributed throughout different tissues and organs, potentially enhancing overall micronutrient intake.

A distinctive feature of edible insects is the presence of chitin, a β-(1 → 4)-linked polymer of N-acetyl-D-glucosamine that forms the exoskeleton and contributes to dietary fibre content [36,37,38]. Chitin has been associated with potential functional properties, including modulation of gut microbiota and short-chain fatty acid production in experimental models [39,40]. In addition, emerging evidence suggests that gastric chitinase activity and host immune responses to dietary chitin may influence gastrointestinal physiology and metabolic outcomes, indicating that chitin may exert effects beyond those of conventional insoluble fibre [41,42]. However, these functional effects remain largely derived from preclinical studies and require further validation in human populations [4,43,44].

Nevertheless, the bioavailability of micronutrients and the physiological fate of chitin in humans remain incompletely characterized and warrant further investigation [45,46]. While in vitro and animal studies suggest favourable mineral solubility and relatively high protein digestibility, the effects of processing methods, food matrix interactions, and anti-nutritional factors (e.g., phytates and tannins) on nutrient absorption require further clarification [45,46]. Similarly, the digestibility of chitin and its long-term effects on intestinal function, nutrient utilization, and metabolic health require systematic evaluation, particularly through well-controlled human intervention studies [36,47,48]. These gaps highlight the need for integrative nutritional and clinical research to substantiate the functional relevance of insect-derived components.

### 3.3. Comparison with Conventional Protein Sources

The nutritional profile of edible insects is comparable to and in some cases may equal or exceed that of conventional animal protein sources such as beef, poultry, and fish [38,49,50]. Many edible insect species contain all essential amino acids and often exhibit amino acid profiles comparable to those of conventional animal proteins, although the concentrations of specific amino acids vary among species [51]. In addition, insect proteins exhibit favourable digestibility and protein quality indices, although variability has been reported across species and processing methods [52,53]. Taken together, these characteristics suggest that edible insects can be nutritionally competitive alternatives.

A key advantage of edible insects over vertebrate meat is that the entire body is usually consumed, rather than only selected muscle tissues. This means that insect biomass may deliver nutrients from the exoskeleton, digestive tract, reproductive tissues and other organs in addition to muscle-like tissues. As a result, edible insects can provide not only protein, but also lipids, vitamins, minerals and fibre in a single matrix, whereas conventional meats are often less diverse in micronutrient and fibre content because they consist mainly of muscle tissue. This whole-body consumption pattern may contribute to the relatively high micronutrient density reported for several edible insect species compared with commonly consumed muscle-based meat portions [33,51,54].

Table 2 suggests that many edible insect species can provide substantial amounts of protein, unsaturated fatty acids, minerals, vitamins and chitin-associated fibre, although species-specific variation is considerable. However, direct comparison with conventional proteins should be interpreted cautiously because reported values depend on analytical methodology, moisture correction, defatting procedure, species identity and the stage of development examined. In addition, comparing insects with meat, legumes, or cereals is not always straightforward, because these foods differ in tissue structure, nutrient distribution and protein quality metrics. Standardized methodology is therefore essential for meaningful cross-study comparisons.

Table 3 further indicates that several edible insect species exhibit favourable digestibility and protein quality characteristics, although reported values are generally more heterogeneous than those available for well-characterized protein sources such as beef and chicken. Mealworm and cricket proteins, for example, may show good protein quality, but the reported ranges still depend strongly on whether the values were obtained by in vitro or in vivo methods and whether chitin or other matrix components were retained [57]. Variability in protein quality is partly attributable to differences in amino acid composition, particularly the concentrations of sulphur-containing amino acids (methionine and cystine), which are frequently identified as limiting amino acids in edible insects [8]. This is important because many insect protein quality values in the literature are derived from in vitro assays, which can overestimate or underestimate true nutritional utilization. Consequently, comparisons with conventional protein sources should be regarded as indicative unless the same analytical framework was applied across all foods. Representative values for mealworm (*Tenebrio molitor*) and house cricket (*Acheta domesticus*) are presented because these species are among the most extensively studied edible insects and have frequently been used in protein quality assessments.

Although conventional animal proteins generally exhibit less variability than insects, reported digestibility and protein quality values may also be influenced by breed, diet, processing and analytical methodology. Another important difference is that edible insects may be more nutritionally sensitive to production conditions than conventional livestock products. Their protein, fat, vitamin, mineral and bioactive compound composition can change substantially with species, feed substrate, developmental stage, rearing environment and processing method, introducing variability but also creating opportunities for nutritional tailoring through controlled production systems. For example, manipulation of feed substrates has been shown to influence fatty acid composition, micronutrient accumulation and overall nutritional quality. By contrast, conventional animal protein sources are often more standardized in composition because of established production chains and well-defined carcass fractions. Consequently, insects should not be viewed merely as substitutes for meat, but as adaptable biological resources whose nutritional characteristics can be deliberately influenced through husbandry and processing strategies.

Overall, the evidence summarized in Table 2 and Table 3 suggests that many edible insect species are nutritionally competitive with conventional protein sources, particularly when their whole-body composition is considered. Their potential advantage lies not only in protein content, but also in the combined provision of essential amino acids, unsaturated lipids, micronutrients and chitin-associated fibre within a single ingredient matrix. Nevertheless, substantial variability among species, production systems and analytical methodologies remains a major challenge, highlighting the need for harmonized reporting, standardized protein quality assessment protocols and additional in vivo validation to support more robust comparisons with conventional foods.

## 4. Environmental and Economic Benefits of Edible Insects

Edible insects have attracted growing interest as sustainable food sources due to their notable environmental and economic advantages over conventional livestock production systems. These advantages make insect farming a promising approach to tackling challenges associated with climate change, resource scarcity, and food security.

From an environmental standpoint, insect farming is linked to relatively low greenhouse gas emissions. Several edible insect species, including *Tenebrio molitor* and *Locusta migratoria*, have been reported to emit substantially lower levels of carbon dioxide and ammonia than traditional livestock such as pigs and cattle, thereby contributing to a reduced environmental footprint [62]. In addition, insect production systems require considerably less land owing to the small body size of insects and their suitability for vertical and space-efficient rearing systems. Consequently, insect farming may demand significantly lower land area than beef production, making it particularly advantageous in regions with limited arable land [4]. Water use efficiency further strengthens the environmental profile of edible insects, as their production generally requires markedly less water than conventional livestock, thereby alleviating pressure on freshwater resources [63].

Beyond environmental sustainability, edible insect farming offers notable economic advantages. Insects exhibit high feed conversion efficiency, translating into lower input costs and improved production efficiency. For instance, crickets may require less than 2 kg of feed to produce 1 kg of body mass, compared with approximately 5 kg for pigs and up to 10 kg for cattle [63]. Moreover, insects can be reared on a wide range of organic substrates, including agricultural residues and food waste, which supports waste valorization and reduces feeding costs [4]. However, the use of waste-derived or alternative substrates is subject to food safety and regulatory requirements in many regions, and substrate selection must therefore balance economic benefits with product safety and regulatory compliance.

An additional advantage of insect production systems is their nutritional flexibility. Unlike many conventional livestock systems, the nutritional composition of insect biomass can be influenced through manipulation of feed substrates [6]. Recent studies have demonstrated that enrichment of rearing substrates with specific nutrient precursors may enhance the accumulation of proteins, fatty acids, vitamins and minerals in insect biomass, thereby creating opportunities for the production of value-added functional ingredients tailored to specific nutritional requirements.

The use of locally adapted insect species may further improve economic sustainability. Native species are often better suited to regional environmental conditions and may require lower inputs for temperature control, humidity regulation and housing infrastructure, thereby reducing operational costs and improving production efficiency. This may be particularly advantageous for small-scale and decentralized production systems in developing regions.

Collectively, these attributes enhance the economic feasibility of insect farming and support its adoption in decentralized, small scale and resource limited production systems. Nevertheless, the environmental and economic performance of insect farming can vary considerably depending on insect species, feed source, production scale, climate conditions and processing requirements [6,64]. Life-cycle assessment studies have reported variability in sustainability outcomes across production systems, indicating that the environmental benefits of edible insects should be evaluated within specific production contexts rather than assumed universally.

## 5. Edible Insect Species and Their Uses

Edible insects comprise a highly diverse group of species consumed globally for food, feed and increasingly for value-added industrial applications. Their use is shaped by cultural tradition, ecological availability, local processing practices and market development. While some species remain closely linked to seasonal harvesting and traditional diets, others are now entering controlled production systems and commercial supply chains.

### 5.1. Commonly Consumed Edible Insect Species

The most commonly consumed edible insect species differ markedly by region, reflecting both biodiversity and culinary tradition [65]. In Southeast Asia, widely consumed species include crickets (*Acheta domesticus*, *Gryllus bimaculatus*), grasshoppers and locusts (*Locusta migratoria*), silkworm pupae (*Bombyx mori*), bamboo caterpillars (*Omphisa fuscidentalis*), giant water bugs (Belostomatidae) and several beetle larvae and palm weevil species [65,66]. These insects are consumed in fresh, fried, roasted, dried, canned and processed forms, and in many countries, they are integrated into everyday diets, street foods and regional specialty products.

In East Asia, silkworm pupae occupy a particularly important place because they are closely associated with sericulture and are widely sold in fresh, frozen and canned forms [67]. Their importance is not limited to one country; they are consumed across China, Korea, Japan and other silk-producing regions. This makes *Bombyx mori* one of the most commercially visible edible insect species in Asia.

In Africa, commonly consumed insects include mopane worms (*Gonimbrasia belina*), other caterpillars, termites and locusts [68]. These species are valued not only for their nutritional contribution but also for their seasonal abundance and role in food security. In many communities, they are still harvested from the wild, processed by drying, roasting, smoking, or frying, and consumed either immediately or stored for later use.

In Mexico and Central America, entomophagy is highly diverse and strongly embedded in local food culture [69,70]. Commonly consumed insects include grasshoppers, ant larvae, maguey worms and a range of other regional species. In many areas, these insects are sold in markets and used in traditional dishes, reflecting a long-standing culinary history rather than a recent trend.

In South America, insect consumption varies widely by country and ecological zone, with some regions showing strong local entomophagy traditions and others much less [71,72]. In Oceania, indigenous entomophagy practices also remain important in certain areas, although these traditions are often underrepresented in mainstream reviews. This broader global picture demonstrates that edible insect consumption cannot be reduced to a small set of farmed species commonly highlighted in the commercial literature.

Black soldier fly larvae (*Hermetia illucens*) are used mainly in feed applications rather than direct human consumption [73]. Their importance lies in their high bioconversion efficiency, protein yield and value in organic waste valorisation systems. However, commercial relevance should not be confused with cultural importance, because many traditionally consumed insects remain underrepresented in industrial supply chains despite their nutritional and gastronomic significance.

Other species, such as yellow mealworms (*Tenebrio molitor*) and house crickets (*Acheta domesticus*), have become particularly important in commercial insect farming because of their suitability for large-scale production, established safety assessments and growing acceptance in processed food products [74].

**Figure 1:**
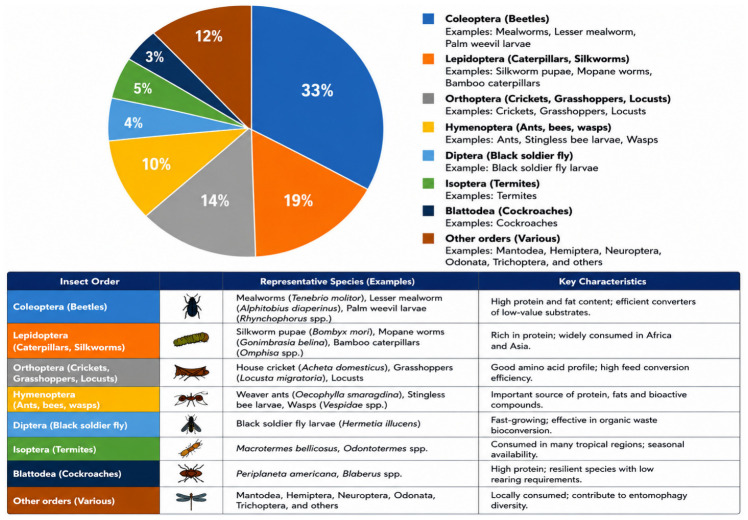
Relative distribution of major edible insect orders reported in human consumption worldwide (%) and representative edible species. Sources: Compiled and adapted from [33,51,65,72,75,76].

The diversity of edible insects extends across multiple taxonomic orders. Although the relative representation of edible species varies among regions and data sources, Figure 1 provides an overview of the principal insect orders reported in human consumption worldwide. Coleoptera, Lepidoptera and Orthoptera account for a large proportion of reported edible species, but Hemiptera, Hymenoptera, Diptera, Isoptera, Odonata, Mantodea, Blattodea and Neuroptera also contribute to global entomophagy. The relative importance of these orders should not be interpreted as uniform across regions, because ecological availability, cultural preferences and historical dietary practices differ substantially between countries and continents.

Overall, commonly consumed edible insect species reflect a combination of traditional dietary practices and emerging industrial applications. A meaningful review must therefore include both widely farmed insects and culturally important wild-harvested species to reflect the true diversity of global entomophagy.

Figure 1 illustrates the taxonomic diversity of edible insects reported worldwide. Although Coleoptera, Lepidoptera and Orthoptera account for a substantial proportion of documented edible species, numerous other orders also contribute to global entomophagy. The relative importance of individual orders varies considerably among regions owing to differences in biodiversity, cultural preferences and harvesting practices.

### 5.2. Regional Patterns of Insect Consumption

Insect consumption is deeply embedded in the dietary traditions of many regions worldwide, although the species consumed and the ways they are prepared differ substantially. In Southeast Asia, edible insects are widely sold as snacks, street foods and preserved products [72]. Commonly consumed species include crickets, grasshoppers, bamboo caterpillars, giant water bugs, palm weevils and silkworm pupae. These products are found not only in local markets but also in supermarkets and specialty retail outlets, showing that insect consumption in the region is both traditional and commercial.

In East Asia, silkworm pupae remain especially important because of their strong association with the silk industry and their broad use in fresh, frozen and canned forms [77]. This makes them one of the clearest examples of a species that links agriculture, food processing and cultural consumption.

In Sub-Saharan Africa, entomophagy remains closely tied to local ecology, seasonal harvesting and food security [78]. Caterpillars, termites, locusts and beetle larvae are consumed in many communities and are often valued as accessible protein sources during periods when conventional animal foods are scarce or expensive. Traditional processing methods such as drying, roasting, smoking and frying remain central to preservation and palatability.

In North Africa, insect consumption is more limited and culturally different from Sub-Saharan patterns [79]. This distinction matters because broad continental labels can hide major differences in ecology, food culture and species availability. The same is true for Asia and Latin America, where internal diversity is substantial and should not be compressed into a single regional category.

In Mexico and Central America, entomophagy is highly developed and culturally embedded. Grasshoppers, ant larvae, maguey worms and other local species are sold seasonally in markets and used in a wide range of dishes. In South America, insect use varies by country and region, with some areas maintaining strong traditional use and others showing more limited adoption. In Oceania, indigenous insect consumption also remains important in certain communities, although it is often overlooked in broad global summaries.

Europe and North America represent emerging markets rather than traditional entomophagy regions [69,71]. Interest in insect foods has increased in recent years because of sustainability concerns, innovation in food processing and growing availability of insect-based ingredients. In these regions, acceptance is generally higher for processed products such as flours, protein bars, pasta and baked goods than for whole insects. As a result, the market is being shaped more by product design, processing and consumer framing than by long-standing culinary tradition.

**Figure 2:**
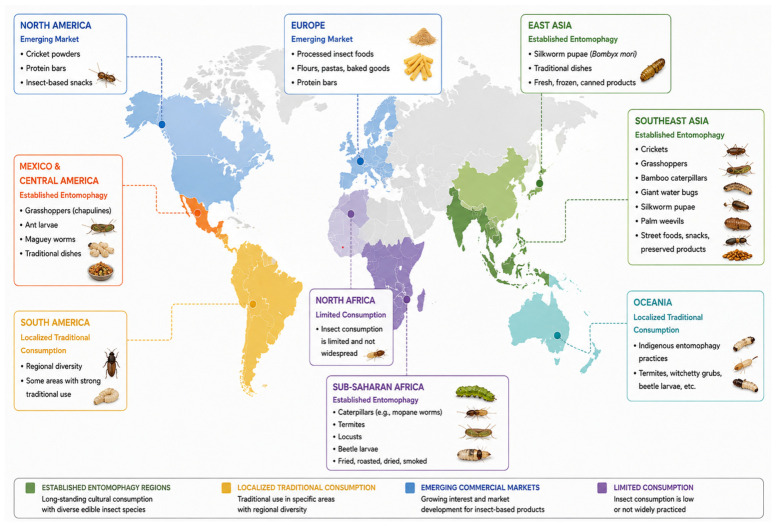
Global distribution of edible insect consumption and representative edible insect groups. Regions are categorized according to established entomophagy traditions, localized traditional consumption, emerging commercial markets and limited consumption areas. Consumption patterns vary considerably within and between regions owing to ecological, cultural and economic factors. Source: Compiled and adapted from [51,54,76,80,81,82,83,84,85]. Regions are color-coded according to their primary entomophagy status as defined in the legend. Shaded areas correspond to the specific regional datasets analyzed, while unshaded (white/gray) areas indicate regions outside the scope of this particular geographical breakdown.

Figure 2 highlights the geographical diversity of entomophagy and demonstrates that insect consumption is concentrated in regions with long-established traditions, particularly Southeast Asia, Sub-Saharan Africa, Mexico and parts of Central America. In contrast, Europe and North America are characterized primarily by emerging commercial markets based on processed insect products [70]. The figure also emphasizes that consumption patterns differ substantially within continents, reflecting local ecological and cultural conditions. This would provide a more accurate and informative representation of global entomophagy.

Overall, the regional patterns of insect consumption show that entomophagy is neither culturally uniform nor geographically limited. A meaningful review should reflect the diversity of species, preparation methods and market systems that define insect use across the world, rather than treating continents as homogeneous consumption zones.

## 6. Processing and Product Development in Edible Insect Farming

The edible insect sector comprises a sequence of interrelated stages, including controlled farming, targeted harvesting, processing, and product development, all of which collectively influence nutritional quality, safety, shelf life, and consumer acceptance of insect-based foods [86]. As commercial production systems continue to expand, increasing emphasis has been placed on developing standardized processing approaches that ensure product consistency while preserving nutritional value [4].

Edible insects intended for human consumption, particularly crickets (*Acheta domesticus*) and mealworms (*Tenebrio molitor*), are commonly reared under controlled environmental conditions in which temperature, humidity, stocking density and feed composition are carefully managed to optimize growth performance and nutrient composition [86]. Feed substrate is especially important because it can influence protein content, lipid composition and micronutrient levels, thereby affecting the nutritional characteristics of the final product. In contrast, black soldier fly larvae (*Hermetia illucens*) are used predominantly in animal feed production and waste bioconversion systems, although interest in their potential food applications continues to emerge.

Harvesting is typically conducted at specific developmental stages to maximize biomass yield, nutritional quality and processing efficiency. For example, mealworms are generally harvested during the larval stage, while black soldier fly larvae are commonly collected before pupation, when protein and lipid yields are often highest [87].

Selection of harvest stage is important because nutrient composition can change substantially throughout insect development, influencing both technological functionality and nutritional value.

Following harvest, insects undergo a range of processing operations designed to improve safety, stability and suitability for food applications. Initial processing often includes cleaning, fasting or gut-emptying procedures to reduce contamination and improve product quality. Subsequent preservation techniques commonly include oven drying, freeze-drying, microwave drying and other dehydration methods that reduce moisture content and inhibit microbial growth [88]. However, processing conditions may also influence nutrient retention, protein functionality, lipid stability and sensory characteristics. For example, high-temperature treatments may enhance microbial safety but can simultaneously affect heat-sensitive vitamins and contribute to lipid oxidation. Consequently, processing methods require careful optimization to balance safety, shelf life and nutritional quality.

Thermal treatments such as roasting, boiling and blanching are frequently applied to improve microbial safety and modify flavour, texture and colour characteristics. Milling of dried insects into powders represents one of the most important technological developments in the sector because it enables incorporation into conventional food matrices while reducing the visual characteristics that often limit consumer acceptance. Processing into powders, protein concentrates and ingredient fractions has therefore become a key strategy for increasing market acceptance in regions where entomophagy is not traditionally practiced.

Product development has expanded considerably in recent years, with insect-derived ingredients being incorporated into a wide range of food products, including bakery items, snacks, pasta, protein bars, beverages and dietary supplements [31,86]. These applications are driven not only by the nutritional value of insects but also by their functional properties, including water-binding capacity, emulsification characteristics and protein enrichment potential. Insect ingredients are also being investigated for use in meat analogues, hybrid meat products and other sustainable food formulations targeted at environmentally conscious consumers.

Among commercially processed edible insects, silkworm pupae (*Bombyx mori*) occupy a particularly important position in several silk-producing countries, including China, Japan and the Republic of Korea [67,89]. As a by-product of the sericulture industry, silkworm pupae are available in large quantities and have long been incorporated into human diets. In addition to fresh consumption, they are widely marketed as frozen, canned and processed products, demonstrating one of the most established examples of industrial-scale edible insect utilization. Their commercial success illustrates how existing agricultural production systems can support the development of insect-based food products while contributing to resource efficiency and value addition.

Despite these advances, several challenges continue to influence large-scale commercialization. Variability in nutritional composition arising from species differences, feed substrates, developmental stages and processing conditions can complicate product standardization. In addition, consumer acceptance remains a significant barrier in many regions, particularly when whole insects are presented as foods. Regulatory requirements, allergenicity concerns and the need for standardized safety assessment protocols further influence market development and international trade. Consequently, future progress in the edible insect sector will depend not only on improvements in farming and processing technologies but also on the development of harmonized quality standards, effective product formulation strategies and consumer-oriented approaches that enhance acceptance of insect-based foods.

## 7. Consumer Acceptance and Challenges of Edible Insects

The successful integration of edible insects into mainstream food systems depends not only on their nutritional and environmental advantages but also on consumer acceptance, regulatory clarity and market development. Although entomophagy is a long-established practice in many parts of Asia, Africa and Latin America, its adoption remains limited in numerous regions where insects are not traditionally regarded as food. Consumer willingness to adopt insect-based products is influenced by a complex interaction of cultural norms, psychological perceptions, food safety concerns, regulatory frameworks and product characteristics. Consequently, acceptance remains one of the most significant challenges facing the expansion of the edible insect sector [90].

### 7.1. Cultural Attitudes and Food Neophobia

Cultural perceptions play a fundamental role in shaping consumer attitudes towards edible insects. In many regions where entomophagy has historically been practiced, insects are regarded as conventional foods and are valued for their sensory qualities, nutritional contribution and cultural significance. In contrast, consumers in many Western countries frequently associate insects with pests, contamination or unsanitary conditions, leading to negative perceptions that hinder acceptance [91].

Food neophobia, defined as the reluctance to consume unfamiliar foods, is widely recognized as a major psychological barrier to insect consumption. Numerous studies have shown that consumers may reject insect-based foods despite being aware of their potential nutritional and environmental benefits [92]. However, food neophobia alone does not fully explain consumer behaviour. Perceived disgust, concerns regarding appearance, texture and taste and the visibility of insect body parts also strongly influence acceptance. Products containing recognizable insect features generally elicit lower acceptance than products in which insects are processed into less visible forms such as powders, protein concentrates or ingredient fractions. This observation has important implications for product development, as consumer acceptance is generally higher for insect-derived ingredients incorporated into familiar foods than for products containing whole visible insects.

Social and cultural influences further affect willingness to consume insect-based foods. Consumer acceptance is often shaped by family traditions, peer behaviour, media exposure and perceived social approval. Repeated exposure to insect-based products and positive consumption experiences have been associated with increased acceptance over time, suggesting that familiarity can reduce psychological barriers [91,92]. In addition, acceptance may vary according to demographic characteristics, educational background, environmental awareness and dietary preferences. These findings indicate that consumer attitudes towards edible insects are determined by a combination of psychological, cultural and product-related factors rather than by food neophobia alone.

### 7.2. Regulatory Frameworks and Food Safety Concerns

Regulatory frameworks governing edible insects vary considerably among regions, reflecting differences in food legislation, risk assessment approaches and market maturity. In the European Union, edible insects intended for human consumption are regulated under the Novel Foods Regulation (EU) 2015/2283 and require safety assessment by the European Food Safety Authority (EFSA) before authorization. Among the insect species authorized for specific food applications are *Tenebrio molitor* (yellow mealworm), *Acheta domesticus* (house cricket), *Locusta migratoria* (migratory locust) and *Alphitobius diaperinus* (lesser mealworm), although additional authorizations may be granted as regulatory evaluations continue to evolve [93,94,95]. These evaluations typically assess nutritional composition, toxicological safety, microbiological risks and allergenic potential.

In the United States, the Food and Drug Administration (FDA) regulates edible insects under the general framework of the Federal Food, Drug and Cosmetic Act (FD&C Act), treating them as “food” when explicitly intended for human consumption and produced under sanitary conditions [96]. The FDA does not maintain insect-specific regulations; instead, insect-based ingredients are evaluated either as Generally Recognized as Safe (GRAS) substances or as food additives requiring premarket review and approval, depending on their intended use and prior food-history evidence [97]. Where sufficient scientific consensus exists, firms may submit GRAS notifications to the FDA, but the costs and data-burden of such evaluations remain a barrier for small-scale producers.

In practice, the FDA expects insect-based products to comply with Good Manufacturing Practices (GMPs), current Good Manufacturing Practice regulations (cGMPs) and hazard control measures analogous to other animal-derived and novel protein foods [98,99]. This includes controls for microbial contamination, appropriate thermal processing and hygienic rearing and processing environments. However, because the traditional regulatory view of insects in the FD&C Act has often treated them as “filth” or unavoidable defects at low levels, there remains ambiguity about how insects are formally classified when intentionally marketed as food, contributing to regulatory uncertainty for the industry [100,101].

In contrast, many low- and middle-income countries where entomophagy is already practiced often lack codified, risk-based regulatory frameworks for commercial insect production, leading to inconsistent quality control, hygiene practices and labelling standards [102,103]. These regional disparities highlight the need for greater harmonization and clarity in international standards, including definitions for edible species, acceptable rearing substrates, microbiological limits and allergen-labelling requirements. Regulatory fragmentation remains one of the major barriers to international trade and market expansion because producers operating across multiple jurisdictions must comply with differing requirements regarding approved species, substrates, processing conditions and labelling practices.

Food safety concerns also constitute a major barrier to consumer acceptance. Key issues include microbial contamination (e.g., from rearing substrates, manure, or cross-contamination in processing), chemical residues (e.g., heavy metals, pesticides), and allergenicity, particularly cross-reactivity between insect and crustacean tropomyosin [104]. Thermal processing (e.g., boiling, roasting, extrusion) can reduce microbial loads and improve safety, but it does not fully eliminate allergenic proteins, which may retain their epitopes through digestion and heating. Transparent labelling of insect-based products, including clear allergen declarations and information about processing methods, is therefore essential to protect sensitized individuals and build consumer trust [105].

Moreover, the absence of universally standardized safety guidelines for edible insects can lead to wide variability in product quality and risk assessment across jurisdictions [102]. Harmonized international frameworks potentially modelled on Codex Alimentarius principles or FAO-WHO guidance could help standardize microbiological limits, allergen-risk assessment protocols, and acceptable rearing substrates, thereby supporting market expansion while safeguarding public health.

### 7.3. Marketing Strategies and Public Education

Effective marketing and education strategies are essential for improving consumer perceptions of insect-based foods. Marketing approaches emphasizing nutritional value, environmental sustainability, and economic efficiency have been shown to positively influence consumer attitudes. Using processed forms such as powders or flours, rather than whole insects, may help lessen visual aversion [106].

Public education initiatives play a complementary role by increasing awareness of the nutritional, ecological, and economic benefits of entomophagy. Science-based communication and transparent information dissemination have been associated with increased willingness to try insect-based products [107]. Furthermore, product development strategies that incorporate insect-derived ingredients into familiar food formats such as bread, pasta, or baked goods can enhance acceptability by aligning novel ingredients with established dietary habits and cultural preferences [108].

**Figure 3:**
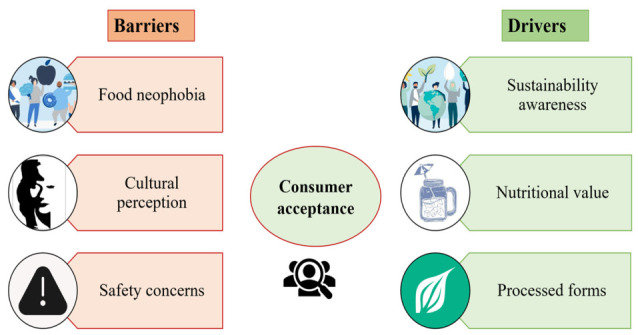
Major barriers and drivers influencing consumer acceptance of edible insects. Consumer willingness to adopt insect-based foods is shaped by interacting psychological, cultural, regulatory, safety and product-related factors, with acceptance generally increasing when insects are incorporated into familiar food formats.

Nevertheless, no single strategy is sufficient to overcome consumer resistance. Successful market development will likely require a combination of product innovation, regulatory transparency, effective communication, repeated consumer exposure and culturally adapted marketing approaches. The key barriers and drivers influencing consumer acceptance of edible insects are summarized in Figure 3.

## 8. Risks, Limitations, and Safety Considerations of Edible Insects

Edible insects offer considerable nutritional and environmental advantages; however, their incorporation into food systems is accompanied by several limitations and safety concerns that require careful evaluation. These include allergenicity, digestibility constraints, microbial and chemical hazards, regulatory uncertainties, ethical considerations and variability associated with production systems. Addressing these challenges is essential for ensuring the safe, sustainable and socially acceptable use of insects as food.

One of the primary health concerns associated with edible insects is allergenicity. Allergic reactions are mainly attributed to tropomyosin, a well-known pan-allergen also present in shellfish and dust mites [109]. Tropomyosin proteins, typically ranging from 35 to 45 kDa, identified in species such as *Acheta domesticus* and *Tenebrio molitor*, exhibit substantial sequence homology (approximately 60–80%) with shrimp allergens, thereby explaining observed cross-reactivity. Reported clinical manifestations include urticaria, respiratory symptoms, and, in rare cases, anaphylaxis in sensitized individuals [110,111,112]. Consequently, individuals with known shellfish or dust mite allergies may represent a higher-risk population for insect-derived foods.

Digestibility-related concerns are also associated with chitin, a structural polysaccharide forming the insect exoskeleton and constituting approximately 2–49% of insect dry weight. Chitin is largely indigestible in humans and has been shown to interact with innate immune pathways, including Toll-like receptor 2 (TLR-2) and nucleotide-binding oligomerization domain-containing protein 2 (NOD2). However, available evidence suggests that chitin itself exhibits limited classical allergenic activity. Instead, residual proteins bound to chitin may indirectly contribute to allergenic responses, whereas purified chitin and its derivative chitosan generally display low immunogenicity [111,113].

Food safety represents another important consideration. Depending on rearing conditions, feed substrate and processing practices, edible insects may harbour microbial hazards, including *Salmonella* spp., *Listeria monocytogenes* and spore-forming bacteria, or accumulate chemical contaminants such as pesticides, veterinary drug residues and heavy metals [104]. However, the magnitude of these risks is highly species-specific and strongly influenced by production practices. Unlike conventional livestock systems, insect farming is still undergoing standardization in many regions, resulting in variability in hygiene management, substrate selection and quality control measures.

An additional challenge arises from the increasing interest in nutritional tailoring of insect biomass. Recent studies have demonstrated that manipulation of feed substrates can enhance the accumulation of desirable nutrients, including essential fatty acids, vitamins and minerals. While this approach offers opportunities to improve nutritional quality, it also highlights the importance of careful substrate selection because undesirable contaminants may likewise be transferred and accumulated within insect biomass. Consequently, nutrient enrichment strategies must be accompanied by rigorous safety assessment and regulatory oversight.

Several processing and management strategies have been proposed to reduce safety risks associated with edible insects. Thermal treatments such as blanching, boiling, roasting and drying can substantially reduce microbial loads, while processing approaches including defatting, enzymatic hydrolysis and protein fractionation may decrease allergenic potential and improve digestibility [114]. In addition, implementation of Good Manufacturing Practices (GMPs), Hazard Analysis and Critical Control Point (HACCP) systems and controlled feeding regimes can reduce the likelihood of chemical contamination and improve product consistency.

Beyond food safety, ethical considerations associated with insect farming are receiving increasing attention. Although insect production is frequently promoted as more sustainable than conventional livestock production, questions remain regarding insect welfare, particularly under intensive farming conditions [6,115]. Scientific consensus regarding insect sentience and welfare requirements remains limited, and issues such as stocking density, environmental conditions and humane harvesting methods continue to be debated. As industrial-scale insect farming expands, the development of science-based welfare guidelines may become increasingly important.

Another limitation concerns scalability and standardization. Nutritional composition, digestibility and safety characteristics may vary substantially among species and according to developmental stage, feed substrate, rearing environment and processing method [55,116]. This variability presents both challenges and opportunities. While it complicates direct comparisons among studies and products, it also provides opportunities to tailor insect biomass for specific nutritional applications through controlled production systems.

Overall, the successful integration of edible insects into food systems will depend not only on their nutritional and environmental advantages but also on effective management of allergenicity, food safety, production variability and consumer concerns. Continued research, harmonized regulations and standardized production practices will be essential for ensuring that edible insects can be utilized safely and sustainably on a larger scale.

The principal food safety hazards associated with edible insects and the corresponding mitigation strategies are summarized in Table 4. Although many of these risks are comparable to those encountered in other animal-derived foods, their magnitude depends strongly on insect species, feed substrate, rearing conditions and processing methods. Consequently, safety management should be considered throughout the production chain, from substrate selection and farming practices to processing, storage and final product distribution.

## 9. Currently Available Insect-Based Protein Products

During the last decade, insect-derived proteins have moved beyond niche food products and are now being used in animal feeds, functional ingredients and specialized nutrition products. This expansion reflects growing interest in alternative protein sources and advances in insect production technologies. Product development has largely focused on transforming insects into ingredients that can be incorporated into familiar food matrices, thereby reducing consumer aversion associated with whole-insect consumption. At the same time, product portfolios differ substantially among regions, reflecting variations in cultural acceptance, regulatory approval and industrial development [120].

Among human food applications, cricket (*Acheta domesticus*) and mealworm (*Tenebrio molitor*) ingredients remain the most widely commercialized in Europe and North America. These species are commonly processed into flours, protein concentrates and powders that are incorporated into protein bars, bakery products, pasta, snacks and dietary supplements [121]. The preference for processed ingredients rather than whole insects reflects consumer studies demonstrating higher acceptance of products in which insect components are not visually recognizable.

However, commercially relevant edible insects are not limited to crickets and mealworms. In several Asian countries, particularly China, Korea and Japan, silkworm pupae (*Bombyx mori*) represent one of the most widely marketed insect-based foods [66,122]. As a by-product of the sericulture industry, silkworm pupae are available in fresh, dried, frozen and canned forms and are utilized both as direct food products and as ingredients in processed foods. Their long-established commercial production distinguishes them from many recently introduced insect-based products and demonstrates that large-scale utilization of edible insects has already been achieved in certain regional markets.

In Latin America, products derived from grasshoppers, ant larvae and other traditionally consumed insects continue to be marketed through local and regional food systems [68,123] while in Africa many edible insects remain predominantly harvested and sold through informal or semi-commercial supply chains. These examples illustrate that the global insect protein sector consists of both modern industrial production systems and traditional market networks, each contributing to food security and dietary diversity in different ways.

Beyond direct human consumption, insect-derived proteins have achieved substantial commercial importance in the animal feed sector. Black soldier fly (*Hermetia illucens*) larvae meal has emerged as one of the most extensively developed insect-feed ingredients due to its efficient bioconversion capacity, favourable amino acid profile and suitability for circular production systems. Its use has expanded particularly in aquaculture, where it serves as a partial replacement for fishmeal and increasingly in poultry, pig and pet food formulations following regulatory approvals in several jurisdictions [124]. In addition to protein, black soldier fly larvae provide functional components such as lauric acid, chitin and bioactive peptides that may contribute to animal health and feed efficiency.

Recent industrial developments have also shifted attention towards fractionated insect ingredients, including protein isolates, lipid fractions, chitin, chitosan and bioactive peptides. These products are increasingly being explored for applications in functional foods, nutraceuticals, sports nutrition, cosmetics and biomedical materials, expanding the commercial relevance of edible insects beyond conventional protein supplementation. Furthermore, advances in insect farming technologies and feed-substrate optimization are creating opportunities to tailor nutrient composition, thereby enhancing specific amino acids, fatty acids, vitamins and minerals in insect biomass through controlled production strategies.

The global insect protein market has experienced steady growth, driven by increasing demand for sustainable protein sources, investment in alternative-protein technologies and supportive regulatory developments in several regions [30]. Nevertheless, market expansion remains uneven, with consumer acceptance, regulatory harmonization, production costs and scalability continuing to influence commercial adoption. Although nutritional and environmental benefits continue to drive interest in the sector, long-term market growth will also depend on improvements in production efficiency, product development, processing technologies and supply-chain integration.

A summary of representative insect-based protein products currently available for human food and animal feed applications is presented in Table 5.

Table 5 demonstrates that commercialization currently focuses on a relatively small number of species despite the much broader diversity of edible insects consumed worldwide. Crickets, mealworms and black soldier fly larvae dominate industrial production because of established rearing protocols, regulatory approvals and supply-chain development. In contrast, species such as silkworm pupae, grasshoppers and palm weevils remain particularly important in regional markets and traditional food systems. This disparity highlights a key challenge for the insect sector: commercially successful species are not necessarily the most culturally important or widely consumed globally. Future product development may therefore benefit from integrating traditional edible species into modern production and processing systems while maintaining regional dietary preferences and market demands.

## 10. Future Prospects and Innovations in Edible Insect Farming

Edible insect farming is expected to continue expanding as advances in production technologies, breeding strategies and product development improve the efficiency and versatility of insect-based systems. Increasing demand for sustainable protein sources, coupled with growing interest in circular bioeconomy approaches, has positioned edible insects as a promising component of future food and feed systems. Recent developments extend beyond simple biomass production and increasingly focus on improving product quality, nutritional value and production efficiency.

Vertical farming and controlled-environment production systems have emerged as important tools for large-scale insect rearing. These systems enable precise regulation of temperature, humidity, photoperiod and diet, thereby improving growth performance, feed-conversion efficiency and production consistency while reducing land requirements and resource use. Integration of Internet-of-Things (IoT) technologies, machine-learning algorithms and real-time monitoring systems further supports automated management of insect colonies by facilitating continuous assessment of environmental conditions, biomass accumulation and production performance in species such as *Tenebrio molitor* and *Hermetia illucens* [127].

Biotechnology and breeding innovations are opening avenues for nutrient-profile optimization, including enhancement of protein quality, essential amino acid balance and micronutrient content (e.g., iron, zinc, B-vitamins). Genome-scale resources and CRISPR-based editing in species such as the house cricket (*Acheta domesticus*) are being explored to improve growth rate, feed efficiency, and disease resistance, and even to reduce allergenicity or tailor lipid profiles for specific applications [128,129]. Selective breeding programs, supported by genomic selection and high-throughput phenotyping, are expected to accelerate genetic gain in commercial insect “crops” and underpin more predictable, scalable production.

An emerging area of particular interest is the targeted enhancement of insect nutritional quality through feed-substrate manipulation and precision nutrition strategies. Recent studies have demonstrated that enrichment of rearing substrates with specific nutrient precursors can increase the accumulation of essential amino acids, beneficial fatty acids, vitamins and minerals in insect biomass. Such approaches enable the production of insect-derived ingredients with tailored nutritional characteristics and improved nutrient bioavailability. Consequently, future insect farming systems may focus not only on maximizing biomass yield but also on designing nutrient profiles that address specific nutritional requirements in human and animal diets.

Automation, robotics and artificial intelligence are streamlining feeding, environmental control, harvesting and processing, thereby lowering labour costs, reducing variability and improving food safety and traceability. Automated conveyor-based rearing, vision-guided grading and closed-loop climate control are already deployed in large-scale black soldier fly and mealworm facilities, helping to meet the rising demand for insect-based protein in aquaculture, poultry and pet-food sectors. Market analysts project the global edible insect market to grow from around USD 1.2–1.4 billion in 2024–2026 to several billion USD by 2030–2035, reflecting strong interest from investors and agri-food companies in sustainable protein diversification [130].

Beyond bulk protein, insects are being explored for personalized and functional nutrition, offering tailored protein supplements, sports-nutrition products and medical food-type formulations. Hydrolysates and enzymatic digests from crickets, mealworms, silkworms and black soldier fly larvae have yielded bioactive peptides with antioxidant, antihypertensive, antidiabetic, anti-obesity, anti-inflammatory and immunomodulatory properties, many of which act on key enzymes such as angiotensin-converting enzyme (ACE) and α-glucosidase [131,132]. These peptides, often effective at low concentrations and with low toxicity, hold promise for managing metabolic syndrome, diabetes, cardiovascular risk and immune-related conditions, although most evidence remains in vitro or preclinical and requires further in vivo validation and clinical trials.

Insect-derived compounds such as chitin, chitosan, antimicrobial peptides and lipid fractions are also attracting interest for applications in nutraceuticals, pharmaceuticals, cosmetics and biomedical materials. In particular, black soldier fly larvae have been investigated as sources of lauric acid and other bioactive lipids with potential antimicrobial and immunomodulatory properties [133,134]. These developments suggest that the future value of insect production may extend beyond bulk protein generation towards the production of specialized bioactive compounds and high-value functional ingredients.

Despite rapid progress in commercial insect farming, it is important to recognize that a substantial proportion of edible insects consumed globally are still harvested from natural ecosystems rather than produced through intensive farming systems. In many regions of Africa, Asia and Latin America, seasonal collection of caterpillars, termites, grasshoppers and other edible species continues to contribute significantly to local food systems and rural livelihoods. Future development of the edible insect sector will therefore require balancing the expansion of industrial production with sustainable management of wild-harvested resources, biodiversity conservation and preservation of traditional knowledge associated with entomophagy.

Overall, future innovations in edible insect farming are likely to be driven by the integration of precision production technologies, genetic improvement, nutrient-tailoring strategies and novel bioproduct development. However, the successful realization of these opportunities will depend on continued research, regulatory development, consumer acceptance and validation of emerging applications through robust scientific evidence.

## 11. Conclusions

Edible insects have emerged as promising alternative protein sources with the potential to contribute to sustainable food and feed systems. In addition to providing high-quality protein, many species are rich in lipids, vitamins, minerals, and bioactive compounds, while their production can require fewer natural resources than many conventional livestock systems and can support circular-economy approaches through the valorization of organic side streams.

However, the nutritional and functional value of edible insects cannot be generalized. Considerable variation exists among species and is influenced by developmental stage, rearing conditions, feed substrate, environmental factors, and processing methods. This variability highlights the need for standardized evaluation approaches and greater comparability across studies. Furthermore, although growing evidence supports the potential of insect-derived compounds such as bioactive peptides, chitin, chitosan, and antimicrobial molecules, many proposed health benefits remain based primarily on in vitro and preclinical studies and require confirmation through well-designed human investigations.

Recent advances in controlled insect farming, precision feeding, automation, artificial intelligence, and genomic technologies are expanding opportunities to optimize nutritional quality and develop value-added insect-derived ingredients for food, feed, nutraceutical, and biomedical applications. At the same time, consumer acceptance, food safety concerns, and differences among regulatory frameworks remain important barriers to wider adoption.

Importantly, edible insects should not be viewed solely as substitutes for conventional animal proteins. They represent a diverse biological resource capable of supporting sustainable nutrition, food-system resilience, and bio-based innovation. Realizing this potential will require harmonized regulations, robust safety standards, improved consumer engagement, and interdisciplinary research capable of translating scientific advances into safe, acceptable, and scalable applications.

## Figures and Tables

**Table 1:** Protein content of selected edible insects on a dry matter basis (%).

Scientific Name	Common Name	Taxonomic Order	Protein Content (% Dry Matter)
*Acheta domesticus*	House cricket	Orthoptera	55–77
*Gryllus bimaculatus*	Two-spotted cricket	Orthoptera	55–75
*Locusta migratoria*	Migratory locust	Orthoptera	58–77
*Tenebrio molitor*	Yellow mealworm	Coleoptera	38–55
*Rhynchophorus* spp.	Palm weevil larvae	Coleoptera	40–65
*Allomyrina dichotoma*	Rhinoceros beetle larva	Coleoptera	40–55
*Gonimbrasia belina*	Mopane worm	Lepidoptera	55–74
*Gynanisa maja*	Emperor moth caterpillar	Lepidoptera	55–65
*Macrotermes bellicosus*	Termite	Isoptera	20–66
*Macrotermes falciger*	Termite	Isoptera	25–60
*Apis mellifera* (drone brood/pupae)	Honeybee pupae	Hymenoptera	45–65
*Polyrhachis* spp.	Weaver ants	Hymenoptera	45–65
*Hermetia illucens*	Black soldier fly larvae	Diptera	36–64
*Musca domestica*	Housefly larvae	Diptera	45–60
*Belostoma* spp.	Giant water bugs	Hemiptera	40–60
*Periplaneta* spp.	Cockroaches	Blattodea	44–66

Values are expressed on a dry-matter basis. Protein content varies depending on species, developmental stage, diet, and processing conditions. Values represent approximate ranges compiled from multiple sources and should be interpreted as indicative rather than definitive. Sources: [2,12,23,24,25].

**Table 2:** Comparative nutritional composition of selected edible insects and conventional protein sources (commonly reported values from the cited literature).

Nutrient Category	Edible Insects (Edible Species)	Conventional Protein Sources (Commonly Reported Values)
Protein content	35–67% protein (range across species; e.g., crickets, grasshoppers, mealworms, black soldier fly)	Beef: ~20% protein; chicken: ~20%; pork: ~17%; soy: ~40–45%; wheat: ~12–15%
Amino acid profile	Generally, contain all essential amino acids and exhibit amino acid profiles comparable to conventional animal proteins; lysine and sulphur-containing amino acid contents vary among species	Beef, chicken and pork provide complete amino acid profiles; soy contains relatively lower methionine levels; wheat is relatively low in lysine
Fats and fatty acids	13–39% fat; contain substantial proportions of MUFA and PUFA, including omega-3 and omega-6 fatty acids; fatty acid composition can be influenced by feed substrate	Soybean: ~18–20% fat; beef, chicken and pork contain varying proportions of saturated and unsaturated fatty acids; soybean oil is rich in PUFA
Carbohydrates and fiber	1–23% carbohydrates; 5–20% dietary fibre, primarily derived from chitin	Meat contains negligible carbohydrate and fibre; soybean and wheat contain higher carbohydrate and fibre levels but do not contain chitin
Minerals	Reported concentrations of Fe, Zn, Cu, Mg, P and Se vary widely among species but may contribute substantially to overall micronutrient intake.	Beef, chicken and pork provide Fe, Zn and P; soy and cereals contribute Mg and P, although mineral bioavailability may be lower in some plant sources
Vitamins	Several species contain vitamin B_12_, riboflavin (B_2_), pantothenic acid (B_5_), biotin and folate, although concentrations vary among species	Meat provides vitamin B_12_ and B-complex vitamins; plant sources such as soy and wheat generally contain little or no vitamin B_12_

Values are presented on a dry-matter basis as approximate ranges. Variations may occur due to species, developmental stage, rearing conditions, feed substrate, and processing methods. Differences in analytical methodologies and reporting standards may limit direct comparability across studies. Abbreviations: MUFA, monounsaturated fatty acids; PUFA, polyunsaturated fatty acids. Sources: [10,12,14,21,22,54,55,56].

**Table 3:** Comparative protein quality indices and amino acid profile of selected edible insects and conventional animal protein sources (per 100 g protein).

Metric (per 100 g Protein)	Beef (Lean)	Mealworm (*Tenebrio molitor*)	Cricket (*Acheta domesticus*)	Chicken Breast
PDCAAS (adults)	0.92	0.64–0.79	0.75–0.89	1.00
DIAAS (young children)	≈1.00	0.59–0.89	0.40–0.92	1.13 (in vitro estimate)
Protein digestibility (%)	94–97	91–99 (in vitro)	79–93 (in vitro)	91–99 (in vitro)
Limiting amino acid(s) relative to adult FAO reference pattern	Methionine	Methionine + Cystine	Methionine + Cystine	None (non-limiting AA pattern)
Lysine (g/100 g protein)	8.1	6.5–7.5	7.0–8.5	8.0

PDCAAS, Protein Digestibility–Corrected Amino Acid Score (truncated at 1.0); DIAAS, Digestible Indispensable Amino Acid Score. Values are expressed per 100 g protein and are based on the amino acid reference pattern for adults and young children [58]. Protein quality indices for insects are largely derived from in vitro assessments and may differ from in vivo estimates. Reported values may vary according to species, developmental stage, rearing conditions and processing methods, including drying, defatting and chitin removal. Values identified as in vitro estimates should be interpreted cautiously when comparing insect and conventional protein sources. Direct comparison of DIAAS values should also be interpreted cautiously because reported values may originate from different experimental approaches and datasets. Sources: [15,16,57,59,60,61].

**Table 4:** Major food safety hazards and mitigation strategies in edible insect production and processing.

Risk Category	Processing/Control Strategies	Remarks
Microbial contamination	Blanching, boiling, roasting, drying, hygienic processing practices	Reduces microbial load and improves product safety; effectiveness depends on species and processing conditions
Allergenicity/chitin	Defatting, enzymatic hydrolysis, protein fractionation, allergen labelling	May reduce allergenic potential, but complete elimination of allergenic proteins is unlikely
Chemical hazards	Controlled feeding substrates, washing, GMPs, HACCP implementation, routine monitoring	Minimizes accumulation of pesticides, heavy metals and other contaminants
Storage-related spoilage	Refrigeration, vacuum packaging, modified-atmosphere packaging, moisture control	Enhances shelf life and reduces microbial growth during storage
Production variability	Standardized rearing conditions, controlled diets and traceability systems	Improves consistency in nutritional quality and safety characteristics

The effectiveness of mitigation strategies may vary depending on insect species, developmental stage, feed substrate, processing conditions and storage environment. Hazard management should therefore be integrated throughout the production chain from rearing to final product development. Sources: [116,117,118,119].

**Table 5:** Representative insect-based protein products currently available for human food and animal feed applications.

Category	Product Type	Main Insect Species	Typical Formulation/Use	Protein Content % (Dry Basis)
Human food products	Cricket flour/powder	*Acheta domesticus*	Protein bars, pasta, baked goods, snacks, tortillas	~60–70
	Mealworm-based foods	*Tenebrio molitor*	Snacks, meatballs, bakery items	~50–65
	Grasshopper-based foods	*Locusta migratoria, Schistocerca* *gregaria*	Chips, energy bars, snacks	~55–70
	Palm weevil products	*Rhynchophorus* spp.	Traditional foods, snacks and regional specialty products	~45–65
	Silkworm pupae products	*Bombyx mori*	Fresh, frozen and canned products; snacks; flour ingredients; traditional foods in Asia	~50–65
Animal feed applications	Black soldier fly (BSF) larvae meal	*Hermetia illucens*	Aquafeed (salmonids, tilapia, shrimp), poultry, and pig feeds	~40–60
	Cricket-based feed	*Acheta domesticus*	Pet food (dog, cat), aquafeed supplements	~55–65
	Mealworm-based feed	*Tenebrio molitor*	Pet food, poultry, and pig feed supplements	~50–60
	BSF-based functional feed	*Hermetia illucens*	Functional feeds enriched with chitin, lauric acid and bioactive peptides	~40–55 crude protein plus high fat and chitin

Protein content refers to crude protein on a dry-matter basis unless otherwise specified. Cricket flour/powder and mealworm-based foods include commercial formulations marketed as human food or animal feed ingredients. Sources: [121,124,125,126].

## Data Availability

No new data were created or analyzed in this study.
